# Mental rotation abilities of gymnasts and soccer players: a comparison of egocentric and object-based transformations. An exploratory and preliminary study

**DOI:** 10.3389/fpsyg.2024.1355381

**Published:** 2024-06-05

**Authors:** Thomas Jürgen Klotzbier, Nadja Schott

**Affiliations:** Institute of Sport and Movement Science, Department of Sport Psychology and Human Movement Performance, University of Stuttgart, Stuttgart, Germany

**Keywords:** mental rotation, soccer players, gymnasts, perceptual task, motor expertise, spatial ability

## Abstract

**Background and objectives:**

The experience obtained from motor expertise may contribute to and enhance the development of particular visuo-spatial abilities. This exploratory and preliminary study compares the response times of a mental rotation task with egocentric and object-based transformation instructions between soccer players of varying performance levels and gymnasts.

**Methods:**

Fifty-six male participants were grouped based on their sports experience. Soccer-specific novices (SS-N: *n* = 19; age = 15.9 ± 0.87), soccer-specific experts (SS-E: *n* = 17; age = 16.4 ± 0.70), gymnastic-specific experts (GS-E: *n* = 10; age = 16.6 ± 1.71), and gymnastic-specific novices (GS-N: *n* = 10; age = 16.0 ± 1.63) were recruited to perform a perceptual task (recognition of soccer-specific poses) and mental rotation tasks with different stimuli (soccer-specific poses, cubes, line-drawings of hands, letters).

**Results:**

During the perceptual task with instructions on egocentric transformation and soccer-specific poses, we observed that gymnasts had longer response times than soccer players. Our findings also suggest that experts correctly identified most of the poses in terms of accuracy. In the mental rotation task with object-based transformation, gymnasts processed all stimuli, even the soccer-specific poses, more accurately than both soccer groups.

**Conclusion:**

Our results suggest that gymnasts’ motor expertise plays a role in their performance on mental rotation tasks involving both egocentric and object-based transformations, regardless of the stimuli presented.

## 1 Introduction

Mental rotation, the ability to mentally represent and manipulate objects, is crucial in daily situations requiring spatial reasoning (Uttal et al., 2013b). Spatial orientation and imagination are essential skills in various professions, including car mechanics, electricians, graphic designers, and physicians (Ha and Fang, 2016; Sorby et al., 2021). Spatial cognition and appropriate motor skills are essential for athletic performance in a sport-specific context (Moreau et al., 2011). Especially in ball games such as soccer or basketball, spatial cognitive abilities are essential to anticipate actions from the offense or defense from different views (Mann et al., 2007; Voss et al., 2010; Ben Mahfoudh and Zoudji, 2022).

Spatial perception, spatial visualization, and mental rotation are recognized as key components of spatial cognition (Linn and Petersen, 1985). However, more recent research has identified additional distinctions between spatial abilities (Holden et al., 2015; Buckley et al., 2018). The study by Buckley et al. (2018) highlights a lack of precise definition and categorization of spatial abilities, recommending the integration of current research findings to address this gap. Mental rotation describes the ability to manipulate two mentally- or three-dimensional objects with respect to their orientation, i.e., to rotate, mirror, or tilt them (Shepard and Metzler, 1971). The term also refers to a paradigm or a specific experimental design that has become increasingly popular in neuropsychological research in recent years. Shepard and Metzler (1971) first described mental rotation as involved in object recognition. They operationalized it using what is now referred to as the classical mental rotation task and measured the response time required to solve the task. Participants were given “mirror-normal discrimination tasks” (Cooper and Shepard, 1973; Cooper, 1975) in which they compared objects. Then, they were required to decide equality and inequality (same-different judgment), irrespective of a possible angular disparity between the comparison objects. The dependent variables measured were the time from the beginning of the stimulus presentation to the response – usually pressing a button – and accuracy. Therefore, Shepard and Metzler’s tests can be described as chronometric tests.

Shepard and Metzler (1971) studied response times when recognizing unfamiliar objects. Their classical study involved the use of three-dimensional (3D) cube figures. The participants were presented with paired images of these cube figures, which were rotated by different angular degrees either in the depth or image planes. The task was to determine as quickly as possible whether the objects could be merged by rotation or not. Shepard and Metzler demonstrated that the response time depends on the angular disparity between the two cubes. That is, the response time increased linearly with increasing angular disparity. However, when the angular disparity between the target and the comparison figure exceeds 180°, there is no further linear increase in response times. This finding is commonly interpreted to suggest that the mental rotation process is analogous to an actual executed rotation. This post-hoc explanation of the time characteristic is based on data obtained through participant introspection in these experiments. The participants reported that they imagined the object in 3D space and were thus able to rotate the object around any axis.

However, a distinction is made between two transformation strategies in mental rotation tasks: an object-based and egocentric (perspective) transformation, which can induce concise tasks and specific instructions (Zacks et al., 2002). How individuals solve a mental rotation task depends on the type of judgment that needs to be made (Steggemann et al., 2011). The former (object-based) requires same-different judgments with respect to pairs of stimuli, while the latter (egocentric) requires left-right judgments with a single stimulus (Feng et al., 2017). Cohen and Kubovy (1993) defined two criteria that must be met to refer to an object-based mental rotation. The first criterion is a positive slope, meaning that the greater the angular disparity between the two objects being compared, the longer it takes to complete the task. Thus, the correlation between response time and degree of rotation is characterized by a monotonically increasing trend up to 180°. The second criterion is the maximum rotation speed, which must not be exceeded. The upper limit of the response time must remain undetermined until the determinants of the rotation process are known. Cooper (1975) discussed another criterion that best reflects the basic idea of mental rotation. The third criterion is the parity decision: Participants will be assigned to make a parity decision. A mental rotation process only occurs if there is an angular difference between the two objects being compared. Participants must determine whether the two objects are identical.

Numerous studies suggest a significant connection between mental rotation and spatial thinking, with motor abilities playing an essential part in both areas (Moreau et al., 2011). Mental rotation is an important aspect of spatial thinking and is associated with mathematical progress and educational achievement (Bott et al., 2023). Additionally, this cognitive skill is closely linked to motor abilities, as evidenced by the impact of motor limitations on mental rotation in children (Krúger and Krist, 2009). The link between spatial cognition and motor skills is further highlighted by the relationship between motor skills and executive functions, which are vital for spatial problem-solving (Stuhr et al., 2020). Theories of embodied perception emphasize the role of interaction between action and cognition (Kiefer and Trumpp, 2012). Several studies have shown that there is a correlation between perceptual ability and motor expertise within the respective domain. Blake and Shiffrar (2007) argue that motor expertise plays a crucial role in the perception of human movement. Markman and Brendl (2005) found that the movement-compatibility effect is influenced by self-representation in space, highlighting the complex interplay between perceptual and motor representations. Beilock (2008) further supported this by demonstrating the pivotal role of sensorimotor experience in embodied cognition. Eskenazi et al. (2009) provided neuropsychological evidence showing that neurological impairments can affect performance and action perception, suggesting a close link between the two. Hohmann et al. (2011) furthered this research by showing that motor expertise can influence action and actor recognition, with experts demonstrating faster reaction times and greater accuracy. These studies underscore the correlation between perceptual ability and motor expertise within their respective domains.

The findings of Proffitt et al. (1995) and Bhalla and Proffitt (1999) also support this approach, indicating that an observer’s physiological potential to climb a hill affects their perception of its slope. This suggests a close link between visual perception and an observer’s motor preconditions and expertise. Expert observers in sports and other domains of visual expertise possess the remarkable ability to quickly and accurately determine the key characteristics of motion (Dicks et al., 2019). This ability is developed through encounters with the same classes of movement, allowing experts to recognize them (Sparrow and Sherman, 2001). Kaltner et al. (2014) suggest that mental rotation performance is not only influenced by motor expertise but also by visual expertise. Furthermore, specific sports training that involves extensive mental rotation ability can significantly enhance mental rotation performance (Moreau, 2012). These findings highlight the strong connection between spatial cognition, motor skills, and sports performance.

Research has demonstrated that individuals with motor expertise have an advantage of this very expertise when performing mental rotation tasks (Jansen and Lehmann, 2013; Habacha et al., 2014, 2022; Schmidt et al., 2016; Weigelt and Memmert, 2021; Kamruddin, 2022). For instance, Habacha et al. (2014) conducted a study comparing table tennis players, who frequently perform and observe rapid hand movements, with soccer players, who lack experts in hand movements, in a mental rotation task using their hands. The authors found that table tennis players exhibited faster mental rotation of their hands and had lower response times for object-based transformations. This study highlights the embodied nature of the mental rotation task of hands by showing selective effects of motor expertise. The study by Jansen and Lehmann (2013) examined the effects of motor expertise on mental rotation tasks involving cube figures and human poses. The study included 40 participants in each group: soccer players, gymnasts, and non-athletes. The study found that all participants had a higher mental rotation accuracy for human poses than cubes. In addition, gymnasts demonstrated better mental rotation performance than non-athletes. Only gymnasts who had practiced rotation movements around the three axes performed better in the mental rotation task, irrespective of the type of stimuli. Soccer players did not perform statistically better than non-athletes. In their meta-analysis, Voyer and Jansen (2017) examined the moderating effects of the relationship between motor expertise and performance on spatial tasks. The authors showed that concerning the type of sport, ball sports have only a small effect, while gymnastics has a medium effect on mental rotation abilities. It is important to emphasize that the study grouped gymnasts and dancers into one gymnastics category. However, when gymnasts and dancers were analyzed separately, very different effect sizes emerged. Gymnasts alone exhibited a high effect size (Cohen’s d) of 0.516 (95% CI = 0.184, 0.938), whereas dancers showed a very small effect of *d* = 0.057 (95% CI = −0.396, 0.509). Weigelt and Memmert (2021) investigated the extent to which the expertise of basketball players accounts for differences in performing mental rotation tasks. The authors observed better mental rotation performance in experts compared to novices.

Previous studies have neglected whole-body rotations and more complex movements (e.g., symmetrical or asymmetrical arm, leg, trunk, or head movements in certain sport-specific skills) in mental rotation tasks (Heinen, 2013). Heinen, therefore, poses the question of whether more complex whole-body rotations have similar effects on mental rotation performance as (relatively simple) hand movements or human line drawings/poses.

Therefore, in the present exploratory and preliminary study, we used the images of body postures that occur in sports movements (soccer-specific skills/poses) rather than regular movements (e.g., arm stretching) to understand better the interplay between sports expertise and the egocentric transformation in mental rotation tasks. Unlike Jansen and Lehmann (2013), we examine soccer players and gymnasts; however, we used more complex whole-body soccer-specific human poses and additional stimuli to compare the two groups directly in one study.

Thus, in the first perceptual task with soccer-specific poses, we want to 1) examine whether soccer-specific poses are egocentrically transformed and show a linear trend between reaction time and angular disparity. If we do not observe a linear trend, we can assume an egocentric transformation and rule out the possibility of a mental rotation process. A key aspect of this perceptual task is to 2) investigate whether soccer players can identify soccer-specific poses faster and more accurately than gymnasts due to their greater familiarity with these types of stimuli. In a second mental rotation task with object-based transformations (poses, cubes, hands, letters), we will 3) investigate whether there is a linear trend (a very well-established finding) between response time and angular disparity when mentally rotating these stimuli. This approach allows us to test whether these stimuli are indeed subject to mental rotation. Our central objective is to 4) analyze whether experts in gymnastics or soccer players can mentally rotate object-based, soccer-specific poses faster and more accurately than their respective novices.

We hypothesized that soccer players would recognize soccer-specific poses more quickly than gymnasts in the perceptual task with egocentric transformation, while the experts would perform best. We expect a linear trend for the mental rotation tasks with object-based transformation, especially for cubes, with cube figures taking the longest to make a parity decision (see Jansen and Lehmann, 2013). We expect that gymnasts will recognize soccer-specific poses faster when they are presented rotated by different degrees around the X, Y, and Z-axes and when a parity decision (same-different judgement) is required.

## 2 Materials and methods

### 2.1 Participants

All participants were recruited at sports clubs through telephone requests by the investigator. The soccer experts (SS-E group) were male players from the German Youth Bundesliga. The soccer novices (SS-N group) were recruited from various soccer clubs in the district league (7th league, Baden-Württemberg). The gymnastics groups (GG-E, GG-N) were recruited from several clubs in the Baden-Wuerttemberg region, where gymnastics is mainly performed at the national and regional levels. Participants were included if they agreed to participate in the study. In this regard, it is a random selection of participants within the groups described above. Athletes had to be in good health and actively trained at the specified national and regional levels to be eligible.

Participation in the exploratory and preliminary study was voluntary, and participants received no financial compensation. They gave their written and verbal informed consent to participate in the study and were informed of the content and procedure of the study. None of the participants had previously participated in an experiment involving mental rotation tasks.

### 2.2 Stimulus material for the perceptual task with egocentric transformation

**FIGURE 1:**
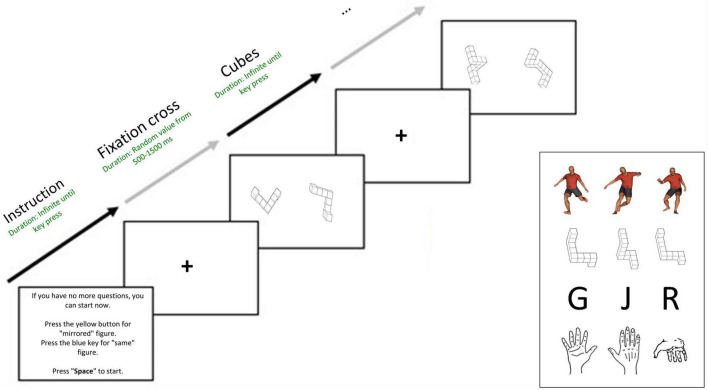
Sequence of the mental rotation experiment in E-Prime^®^ using the example of the task with cubes.

Smith Micro’s Poser^®^ Version 8 3D graphics software was used to design the soccer-specific poses (see Figure 1 and Supplementary Material A). These poses, familiar to soccer players, show (1) an instep drive, a typical attacking position used to score a goal; (2) a cross ball, also called a fly ball; and (3) an inside kick, as used in passing.

The three poses, their mirroring, and the rotations (80°, 160°, 240°, and 320°) around the Z axis (vertical axis) were used (see Supplementary Material A; 3 poses × 2 reflection × 4 rotation angles × 1 axis “Z”). Thus, one cycle contains 24 poses/trials. These 24 poses were presented in three cycles: 24 poses × 3 cycles = 72 poses/trials. Participants were instructed to decide whether the soccer-specific pose depicted a human figure trying to kick the ball with the right or left leg as quickly as possible.

### 2.3 Stimulus material for the mental rotation task with object-based transformation

The mental rotation tasks’ stimuli consisted of identical or mirrored figures presented simultaneously and side by side. In all mental rotation tasks, participants had to follow an object-based instruction and decide as quickly as possible whether the two presented stimuli were the same or different, making as few errors as possible, regardless of their rotation in space (80°, 160°, 240°, and 320° in the X, Y, and Z axes).

**FIGURE 2:**
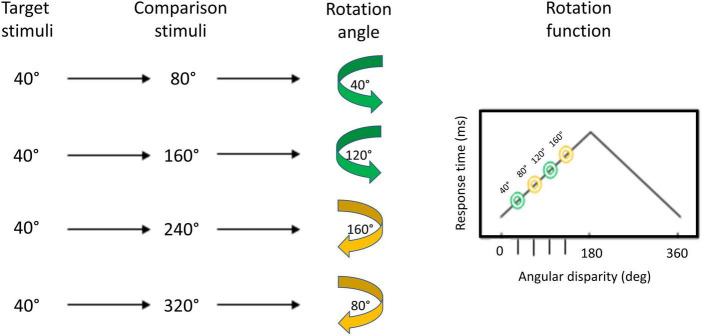
Schematic representation of the rotation function and the angles to be mentally rotated. The green (counterclockwise) and yellow (clockwise) arrows above the angle to be rotated indicate the shortest path of a rotation. For a 320° position of the comparison figure, one of the two figures (target or comparison figure) must be rotated by 80°.

In the mental rotation tasks, soccer-specific poses, cube figures, letters, and line drawings of hands were used (see Figure 1 and Supplementary Materials B–E). The different poses and cubes were presented in a randomized order and rotated around the X (horizontal), Y (transverse), and Z (vertical) axes. The letters and hands were also randomized and presented with rotations around the Z-axis only. The target figure of all stimuli (poses, cubes, hands, letters) was always presented in a 40° position (see Supplementary Materials B–E). The comparison figures were presented simultaneously in 80°, 160°, 240°, and 320° positions, side by side with the target figure. Figure 2 shows the angle that must be rotated to transform the comparison figure into the target figure. The angles selected in this way can be used to determine whether mental rotation is involved in processing the stimuli. If RT of 80° < RT of 320° < RT of 160° < RT of 240°, then mental rotation can be assumed due to the linear function (see Figure 2). This function also implies the ability to identify the shortest path for mental rotation (Takano, 1989).

We used the soccer-specific poses already used in the simple perceptual task shown in Figure 1 for the mental rotation task. The target pose and a comparison pose with different angular positions were presented simultaneously and side by side (see Supplementary Materials B, F; 3 poses × 2 reflection × 4 rotation angles × 3 axis “X, Y and Z”). A cycle of soccer-specific poses thus contains 72 trials. These 72 trials were presented in three cycles: 72 trials × 3 cycles = 216 trials.

The cubes for this exploratory and preliminary study were taken from the “Mental Rotation Stimulus Collection” by Peters and Battista (2008). This collection consists of 16 different stimuli, their reflections, and orientations from 0° to 360°, each with 5° angle variations. Three cubes from this collection were randomly selected (see Figure 1). An example of a cube with four orientations around the X–axis can be found in Supplementary Material C (3 cubes × 2 reflections × 4 rotation angles × 3 axes “X, Y and Z”). A cycle with cubes thus contains 72 trials. These 72 trials were presented in three cycles: 72 trials × 3 cycles = 216 trials.

The letters used are G, J, and R and their reflections (see Figure 1). Other studies have already used these letters (Cooper and Shepard, 1973; Kail et al., 1980). These letters are 2D and only presented in rotation around one axis (Z-axis) (see Supplementary Material D; 3 letters × 2 reflection × 4 rotation angles × 1 axis “Y”). A cycle with letters thus contains 24 trials). These 24 trials were presented in three cycles: 24 trials × 3 cycles = 72 trials.

The hands selected were those used in the experiments by Parsons (1987) and, a few years later, by Lameira et al. (2008). These are line drawings of three different hand positions (palm, back of the hand, and wrist; see Figure 1). These hands are 2D and presented only in rotation around one axis (Z-axis) (see Supplementary Material E; 3 hands × 2 reflection × 4 rotation angles × 1 axis “Y”). A cycle with hands thus contains 24 trials. These 24 trials were presented in three cycles: 24 trials × 3 cycles = 72 trials.

### 2.4 Experimental setup and procedure

The experiment was conducted using a notebook with a 17.3” display and the presentation software E-Prime^®^. For this purpose, a program structure was developed in E-Prime^®^ to run the blocks and stimuli sequentially and to record the response times and the response accuracy. The blocks were presented in a randomized order, similar to the corresponding stimuli. The respective stimuli with a size of 8cm were presented on a white background. The participants used their index fingers to type their answers on two keys of the notebook keyboard. The answer key “x” (German keyboard layout) positioned on the left and the answer key “m” placed on the right were each color-coded (yellow for “same” or “left” and blue for “different” or “right”; depending on the task to be performed). The choice of the index finger was motivated by the need for standardized data collection and response time recording. Selecting the index finger could potentially minimize variability across participants (Yang et al., 2003), given that it is a commonly preferred and frequently used finger for various tasks (Cavina-Pratesi and Hesse, 2013). This approach aims to promote more uniform data collection across the study population. Annett and Annett (1979) illustrated that in tasks involving simple and choice reaction times, responses tended to be faster when alternating between fingers on different hands rather than those on the same hand. Furthermore, no discernible effects were noted based on hand preference or gender. Conversely, some researchers employ the left and right mouse clicks as response options (Kaltner et al., 2014; Pietsch et al., 2019). The experiment was conducted in a quiet room. The rooms were kept dark during the test phase to avoid light reflections on the screen.

The task was to respond to the stimuli as quickly as possible by pressing the correct key. The participants completed a short practice block after reading the instructions in E-Prime^®^. The practice blocks were designed to familiarize the participants with the stimuli and tasks and consisted of 10 practice trials for each stimulus and task. The test was not started until the practice block was completed with an accuracy of 66.6% (2/3 correct responses). If 66% was not achieved, the practice block was repeated. A short 5-min break was taken between the test blocks. The total test duration was approximately 60–80 min, depending on individual response times.

### 2.5 Statistical analyses

E-DataAid^®^ was used for initial data inspection. Using E-Basic, the programming language underlying E-Prime^®^, a program command was written to filter the response times of correct trials (ms) and accuracy (%). Similar to Jost and Jansen (2020), reaction times were analyzed for rotated trials but not for trials in which the comparison stimuli were mirrored. Data was then analyzed using SPSS version 29.0 (SPSS Inc., Chicago, Illinois). R was used to graph the results (Wickham, 2016; R Core Team, 2023).

First, we examined the response times and accuracy for missing values, normality of distributions (tested by Kolmogorov–Smirnov tests), and the presence of outliers. An alpha level of 0.05 was used for all statistical tests. Group comparisons for continuous variables (such as age and BMI) were assessed using ANOVAs; categorical demographic variables were compared using Chi-Square. Effect sizes for all ANOVAs were reported using Partial Eta Square (η^2^*p*) (Lakens, 2013), with a small effect defined as 0.01, a medium effect as 0.06, and a large effect as 0.14 (Cohen, 1988).

A power analysis (Superpower; Caldwell and Lakens, 2019) was conducted on a 2x2x4 ANOVA with repeated measures design with 10 participants per cell (16 cells in our design). Assuming a medium effect size of *f* = 0.2828 (according to Brysbaert (2019), p. 7), an effect size of *d* = 0.4 is considered interesting, as it has practical relevance; f = (0.4 / √2), a standard deviation of 1.0, and a correlation of 0.2 [an effect size of *d* = 0.4 corresponds to a correlation of *r* = 0.2; Brysbaert (2019), p. 7], and with an alpha of 0.05, a power of 100% was found for the main effect of group, a power of 100% for the main effect of expertise, a power of 96.3% for the main effect of angle, a power of 6.1% for the interaction effect of group × expertise, 4.7% for the interaction effect of group × angle, 6.2% for expertise × angle, and 5.9% for the three-way interaction of group × expertise × angle. Due to the limited power for detecting interaction effects within our analyses, we can only provide reliable statements regarding the main effects, which aligns with the recommendation made by Brysbaert (2019).

We also conducted additional analyses to assess the impact of speed-accuracy trade-offs. Specifically, we examined the response time data to determine whether faster responses were associated with decreased accuracy, which could suggest a trade-off. For this purpose, bivariate correlations were calculated between each stimulus’s average reaction times and the respective stimulus’s average accuracy.

#### 2.5.1 Analysis of the perceptual task with egocentric transformation

Bivariate correlations with the variables reaction time and rotation angle (40°, 80°, 120°, and 160°; see Figure 2) were calculated to show a linear trend in the egocentric-based transformation (same - different judgment) between angular disparity and reaction time. The larger the angular disparity between the two objects of comparison, the longer the response times. A monotonically increasing trend characterizes the relationship between response time and degree of rotation. Mental rotation can be assumed for linear trends and monotonic slopes (Cohen and Kubovy, 1993). Studies indicate that both “live matches” and “TV matches” impact the perception and performance of soccer skills (Bruland and van der Kamp, 2012), aligning with embodied perception theory (Shiffrar and Heinen, 2011, 2015). Pizzera and Raab (2012) delved deeper into the influence of motor and visual experience on decision-making among sports officials, emphasizing their significant role in perception. These findings emphasize the intricate connection between live/TV matches and sports-specific skill perception, with embodied perception theory playing a central role. For this reason, these factors were included as covariates to control for their confounding influence.

A 2 (sports: gymnastics and soccer) × 2 (expertise: experts and novices) × 4 (angle: 80°, 160°, 240°, and 320°) repeated measures ANCOVAs were performed for the mean response times at all rotation angles with each stimulus as the dependent variable and group as the fixed factor with “live matches” and “TV matches” as covariates (since not only motor expertise but also visual experience plays an important role in mental rotation performance; Kaltner et al., 2014) to examine group differences in the processing of the perceptual task with soccer-specific poses.

#### 2.5.2 Analysis of the mental rotation task with object-based transformation

Bivariate correlations with the variables reaction time and rotation angle (40°, 80°, 120°, and 160°; see Figure 2) were calculated to show a linear trend in the object-based transformation (same - different judgment) between angular disparity and reaction time.

A 2 (sports: gymnastics and soccer) × 2 (expertise: experts and novices) × 4 (angle: 80°, 160°, 240°, and 320°) repeated measures ANOVA was performed to examine the group difference in reaction times of the mental rotation task with soccer-specific poses, cubes, letters, and hands (within-subject factors).

## 3 Results

### 3.1 Sample characteristics

Fifty-six participants (all male, age = 16.2 ± 1.12) voluntarily participated (see Table 1 for participant characteristics). The participants were divided into the following groups depending on their sports activity: Concerning soccer-specific sports experience, two experimental groups were formed. The soccer-specific expert (SS-E) group (*n* = 17; age = 16.4 ± 0.70) is playing for the B - junior team of a Bundesliga club, with a high soccer-specific expertise. The second group of soccer players (*n* = 19; age = 15.9 ± 0.87) is called soccer-specific novices (SS-N) since they have a significantly lower training volume and play in a lower league. The participants were asked to complete a questionnaire about their sports biography to obtain these characteristics. In addition, a third and a fourth group with gymnastics-specific experts (GS-E; *n* = 10; age = 16.6 ± 1.71) and gymnastics-specific novices (GS-N; *n* = 10; age = 16.0 ± 1.63) participated in the exploratory and preliminary study. The gymnasts competed mainly in the Swabian Gymnastics Federation.

**TABLE 1:** Characteristics of SS-E, SS-N, GS-E, and GS-N, including mean values (standard deviation) and an inferential statistical comparison of the groups.

	SS-E	SS-N	GS-E	GS-N	Stat. analyses
	(*n* = 17)	(*n* = 19)	(*n* = 10)	(*n* = 10)	
Age (years, SD)	16.4 (0.70)	15.9 (0.87)	16.6 (1.71)	16.0 (1.63)	*F*(3,51) = 0.85, *p* = 0.472, η^2^_p_ = 0.048
BMI (kg/m^2^)	22.6 (1.37)	21.4 (1.85)	20.6 (2.64)	20.9 (2.65)	*F*(3,49) = 3.17, *p* = 0.032, η^2^_p_ = 0.162
Live soccer matches (%)					*CHI*^2^(9) = 54.6, *p* < 0.001
None	0	0	80.0	60.0	
1 match	0	0	10.0	30.0	
2–4 matches	17.6	36.8	10.0	10.0	
More than 4 matches	82.4	63.2	0	0	
TV soccer matches (%)					CHI^2^(9) = 40.9, *p* < 0.001
0 min	0	0	50.0	50.0	
1–60 min	11.8	10.5	40.0	50.0	
61–120 min	23.5	31.6	10.0	0	
More than 120 min	64.7	57.9	0	0	
Age at onset with regular training (years, SD)	5.29 (1.05)^§^	7.68 (2.65)^ŧ^	6.40 (1.17)	6.40 (1.17)	*F*(3,52) = 5.30, *p* = 0.003, η^2^_p_ = 0.234
Training duration per week (min)	763 (90.3)^§^^,^^$^^,^^#^	267 (63.9)^ŧ^^,^^$^	569 (183)^ŧ^^,^^§^^,^^$^	340 (156)^ŧ^^,^^$^	*F*(3,52) = 59.3, *p* < 0.001, η^2^_p_ = 0.774
Greatest sports success (all sports)					CHI^2^(8) = 31.8, *p* < 0.001
No competitive activity	0	5.3	0	0	
Local competitions	0	10.5	0	0	
Regional competitions	5.9	52.6	70.6	80.0	
National competitions	23.5	26.3	20.0	10.0	
International competitions	70.6	5.3	10.0	10.0	

ŧ significant difference to SS-E (*p* < 0.05);

§ significant difference to SS-N (*p* < 0.05);

$ significant difference to GS-E (*p* < 0.05);

# significant difference to GS-N (*p* < 0.05).

Concerning the age of starting regular training, there was an expertise main effect (*F*(1,52) = 6.65, *p* = 0.013, η^2^_p_ = 113) and a sports × expertise interaction effect (*F*(1,52) = 4.75, *p* = 0.034, η^2^_p_ = 0.084). The SS-E started regular training significantly earlier than all other groups. Concerning the amount of training, the expertise groups differed significantly (*F*(1,52) = 120, *p* < 0.001, η^2^_p_ = 698). Experts had a higher training volume compared to the novices. The difference in training volume between experts and novices was higher for the soccer players than for the gymnasts (*F*(1,52) = 16.4, *p* < 0.001, η^2^_p_ = 239).

In addition to active sports, the additional involvement with soccer is also of particular interest. Overall, soccer players watch more live games and more games on TV compared to gymnasts. No expertise effect was found.

### 3.2 Results for the perceptual task with egocentric transformation

**FIGURE 3:**
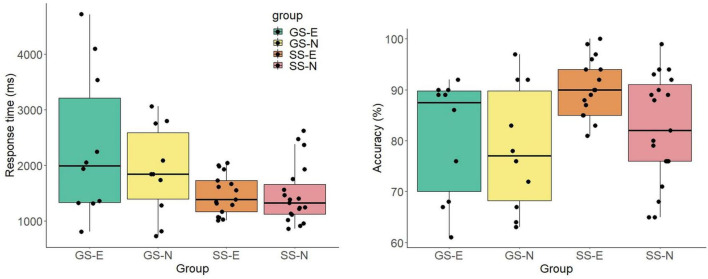
RT and accuracy (mean and standard error) as a function of angular disparity for mental rotation of soccer poses with egocentric transformation.

The bivariate correlation between angular disparity and response time (*r* = −0.08) showed no significant linear trend (*p* = 0.249). In contrast, the bivariate correlation between angular disparity and accuracy showed a significant linear trend (*r* = 0.179, *p* = 0.07). The correlations examining the Accuracy-Speed trade-offs reveal no significant relationships (*r* = 0.01, *p* = 0.997). An ANCOVA for response accuracy, controlled for live and televised soccer games, and the independent variables sports and expertise showed no significant effects for the main effect angular disparity or the interactions with the sports group and expertise group (see Figure 3 right graph). Only the between-subjects effect expertise showed an overall significant effect (*F*(1,48) = 4.31, *p* = 0.044, η^2^_p_ = 0.086), with experts (86.0 ± 10.2) achieving higher overall accuracy than novices (80.1 ± 10.0). An ANCOVA for reaction time, controlled for live and televised soccer games, and the independent variables sports and expertise showed a significant interaction for angular disparity by sports (*F*(2.30,110) = 8.03, *p* < 0.001, η^2^_p_ = 0.143). RTs did not differ in angular disparity for soccer players but for gymnasts (see Figure 3 left graph). In addition, there was a significant interaction between angle disparity and watching live soccer matches (*F*(2.30,110) = 4.95, *p* = 0.006, η^2^_p_ = 0.093). Overall, people watching two or more games were equally fast for different angles. In contrast, those watching only one or no game were slower overall and showed higher RTS with increasing angle disparity. The between-subjects effect sports showed an overall significant effect (*F*(1,50) = 8.71, *p* = 0.005, η^2^_p_ = 0.154), with soccer players (1479 ± 465) reacting faster than gymnasts (2119 ± 1091).

### 3.3 Results for the mental rotation task with object-based transformation

**FIGURE 4:**
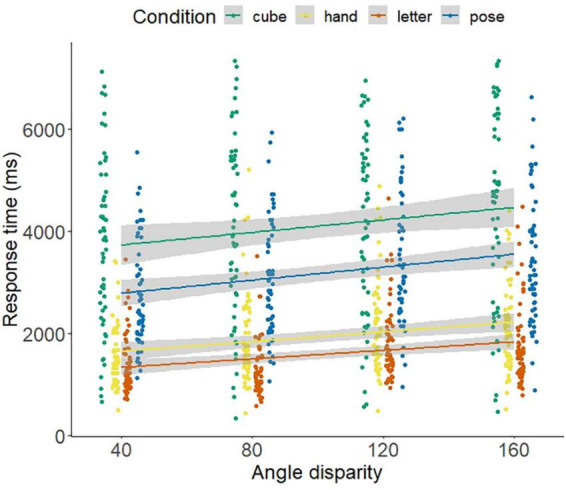
Linear trend between the response time (ms) and the angle to be rotated (disparity) separated by stimuli and groups. Linear fitting cures and individual data points. Angle disparity describes the angle to be rotated up to the target figure.

The bivariate correlations between the angle to be rotated and the response times indicated a linear trend, with *r* = 0.233, *p* < 0.001 for the poses, *r* = 0.190, *p* < 0.001 for the cubes, *r* = 247, *p* < 0.001 for the hands, and *r* = 0.169, *p* = 0.011 for letters (see Figure 4).

**FIGURE 5:**
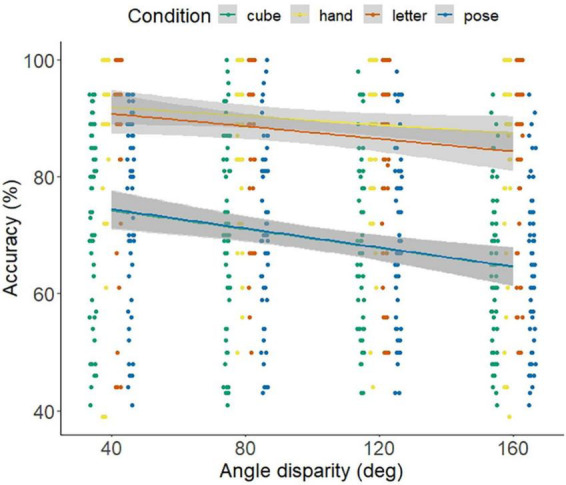
Linear trend between the accuracy and the angle to be rotated (disparity) separated by stimuli and groups. Linear fitting cures and individual data points. Angle disparity describes the angle to be rotated up to the target figure.

The bivariate correlations between the angle to be rotated and the accuracy indicated a linear trend, with *r* = −0.217, *p* = 0.05 for the poses, *r* = −0.216, *p* < 0.05 for the cubes, and *r* = −0.177, *p* = 0.008 for letters. No linear trend for hands (*r* = −0.129, *p* = 0.054) could be observed (see Figure 5). The correlations examining the Accuracy-Speed trade-offs reveal no significant relationships for poses (*r* = 0.252, *p* = 0.061), however a significant correlation for letters (*r* = 0.428, *p* < 0.01), hands (*r* = 0.275, *p* = 0.040) and cubes (*r* = 596, *p* < 0.001).

The 2 (sports: gymnastics and soccer) × 2 (expertise: experts and novices) × 4 (angle: 80°, 160°, 240°, and 320°) for accuracy as well as RT of soccer poses showed a main effect for angle disparity, with the highest accuracy for the 80° condition and the longest RTs for 160° and 240°. No significant interactions between angle disparity and sports or expertise were observed. The between-subjects effect sports showed an overall significant effect (*F*(1,52) = 6.05, *p* = 0.017, η^2^_p_ = 0.104) for accuracy, with soccer players (77.1 ± 16.2) achieving lower overall accuracy than gymnasts (87.3 ± 10.2).

The 2 (sports: gymnastics and soccer) × 2 (expertise: experts and novices) × 4 (angle: 80°, 160°, 240°, and 320°) ANOVA with repeated measures for accuracy, as well as RT of cubes, showed a main effect angle disparity, with the highest accuracy for the 80° condition and the longest RTs for 240°. No significant interactions for angle disparity with sports or expertise were observed. The between-subjects effect sports showed an overall significant effect (*F*(1,52) = 20.0, *p* < 0.001, η^2^_p_ = 0.278) for accuracy, with soccer players (63.8 ± 13.0) achieving lower overall accuracy than gymnasts (79.2 ± 10.3).

The 2 (sports: gymnastics and soccer) × 2 (expertise: experts and novices) × 4 (angle: 80°, 160°, 240°, and 320°) ANOVA with repeated measures for accuracy as well as RT of letters showed a main effect angle disparity with the highest accuracy for the 80° and the 320° condition and the longest RTs for 160° and 240°. Furthermore, there were significant interactions for RT between angle disparity and sports and expertise. Soccer players had significantly higher RTs than gymnasts, especially for the angular degrees 160 and 240; experts, in turn, had higher RTs, especially for the angular degrees 240 and 320. The between-subjects effect sports showed an overall significant effect (*F*(1,43) = 10.2, *p* = 0.003, η^2^_p_ = 0.192), with soccer players (1811 ± 935) reacting slower than gymnasts (1378 ± 594).

The 2 (sports: gymnastics and soccer) × 2 (expertise: experts and novices) × 4 (angle: 80°, 160°, 240°, and 320°) ANOVA with repeated measures for accuracy as well as RT of hands showed a main effect angle disparity, with the highest accuracy for the 80° condition and the longest RTs for 160° and 240°. No significant interactions for disparity with sports or expertise were observed. The between-subjects effect sports showed an overall significant effect (*F*(1,47) = 9.34, *p* = 0.003, η^2^_p_ = 0.175), with soccer players (2060 ± 681) reacting slower than gymnasts (1514 ± 489).

**FIGURE 6:**
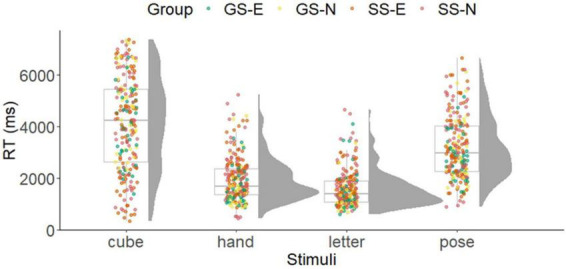
Results of response time in the mental rotation task separated by stimuli and groups. Boxplot with individual data points and density function. GS-E, gymnastic-specific expertise; SS-E, soccer-specific expertise; SS-N, soccer-specific novices.

**FIGURE 7:**
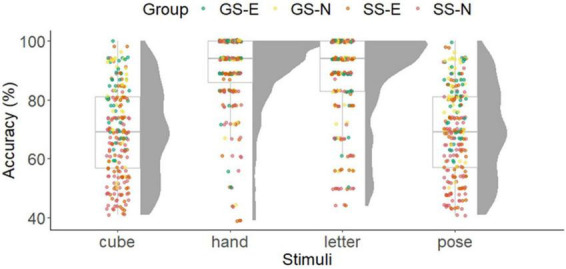
Results of accuracy in the mental rotation task separated by stimuli and groups. Boxplot with individual data points and density function. GS-E, gymnastic-specific expertise; SS-E, soccer-specific expertise; SS-N, soccer-specific novices.

The inferential statistical results of the ANOVAs are presented in Table 2. Figures 6, 7 show the results for RT and accuracy of the mental rotation tasks separated by stimuli and group.

**TABLE 2:** Results of the 4 (group: SS-E, SS-N, GS-E, and GS-N) × 4 (angle: 80°, 160°, 240°, and 320°) ANOVA with repeated measures for response time and accuracy of the mental rotation task with poses, cubes, letters, and hands (mean & SD).

	SS-E (*n* = 17)	SS-N (*n* = 19)	GS-E (*n* = 10)	GS-N (*n* = 10)	angle	angle × sport	angle × expertise	angle × sports × expertise
**Mental rotation with soccer-specific poses**								
RT (ms)	3244 ± 1227	3440 ± 1360	2850 ± 770	3027 ± 1123	*F*(1.91,99.2) = 33.9, *p* < 0.001, η^2^p = 0.395	*F*(1.91,99.2) = 0.04, *p* = 0.957, η^2^p = 0.007	*F*(1.91,99.2) = 0.78, *p* = 0.455, η^2^p = 0.015	*F*(1.91,99.2) = 0.79, *p* = 0.785, η^2^p = 0.004
ACC (%)	77.0 ± 15.9	77.2 ± 17.0	88.5 ± 6.3	86.1 ± 13.3	*F*(2.13,111) = 20.7, *p* < 0.001, η^2^p = 0.285	*F*(2.13,111) = 0.35, *p* = 0.717, η^2^p = 0.007	*F*(2.13,111) = 1.19, *p* = 0.311, η^2^p = 0.022	*F*(2.13,111) = 0.17, *p* = 0.854, η^2^p = 0.003
**Mental rotation task with cubes**								
RT (ms)	3935 ± 2197	4172 ± 1817	3943 ± 1251	4780 ± 1511	*F*(2.58,134) = 38.6, *p* < 0.001, η^2^p = 0.426	*F*(2.58,134) = 2.12, *p* = 0.111, η^2^p = 0.039	*F*(2.58,134) = 0.51, *p* = 0.646, η^2^p = 0.010	*F*(2.58,134) = 2.06, *p* = 0.118, η^2^p = 0.038
ACC (%)	66.3 ± 14.0	61.6 ± 11.9	79.3 ± 9.1	79.0 ± 12.0	*F*(2.58,134) = 18.9, *p* < 0.001, η^2^p = 0.267	*F*(2.58,134) = 1.47, *p* = 0.230, η^2^p = 0.027	*F*(2.58,134) = 0.84, *p* = 0.459, η^2^p = 0.016	*F*(2.58,134) = 1.32, *p* = 0.273, η^2^p = 0.025
**Mental rotation task with letters**								
RT (ms)	1693 ± 525	1622 ± 482	1253 ± 315	1206 ± 335	*F*(2.31,99.1) = 48.9, *p* < 0.001, η^2^p = 0.532	*F*(2.31,99.1) = 3.59, *p* = 0.026, η^2^p = 0.077	*F*(2.31,99.1) = 4.56, *p* = 0.009, η^2^p = 0.096	*F*(2.31,99.1) = 1.35, *p* = 0.264, η^2^p = 0.030
ACC (%)	89.9 ± 10.5	93.3 ± 6.4	92.6 ± 6.7	94.0 ± 5.2	*F*(2.03,86.1) = 12.4, *p* < 0.001, η^2^p = 0.223	*F*(2.03,86.1) = 1.81, *p* = 0.170, η^2^p = 0.040	*F*(2.03,86.1) = 1.67, *p* = 0.194, η^2^p = 0.037	*F*(2.03,86.1) = 1.06, *p* = 0.352, η^2^p = 0.024
**Mental rotation task with hands**								
RT (ms)	2182 ± 707	1965 ± 665	1647 ± 602	1368 ± 290	*F*(2.39,113) = 28.5, *p* < 0.001, η^2^p = 0.378	*F*(2.39,113) = 1.44, *p* = 0.239, η^2^p = 0.030	*F*(2.39,113) = 0.11, *p* = 0.926, η^2^p = 0.002	*F*(2.39,113) = 1.04, *p* = 0.366, η^2^p = 0.022
ACC (%)	93.7 ± 4.0	88.7 ± 12.3	92.1 ± 8.1	95.7 ± 4.0	*F*(3,141) = 4.86, *p* = 0.003, η^2^p = 0.094	*F*(3,141) = 0.27, *p* = 0.846, η^2^p = 0.006	*F*(3,141) = 0.76, *p* = 0.518, η^2^p = 0.016	*F*(3,141) = 0.76, *p* = 0.521, η^2^p = 0.016

RT, response time; ACC, accuracy.

## 4 Discussion

The exploratory and preliminary study aimed to compare the mental rotation performance of gymnasts and soccer players of different expertise levels. Given this study’s limited number of cases, it is advisable to interpret the results with caution. Particularly due to our small sample size and limited statistical power regarding the interaction effects, definitive conclusions cannot be drawn (refer to Brysbaert, 2019, p. 27). This should be considered when interpreting the results and in the subsequent discussion.

Soccer-specific poses were utilized in the initial perceptual task, requiring participants to make right-left decisions. The study assessed whether this task induces an egocentric transformation and whether differences are present among the groups. We found no significant correlation between response time and angular disparity, suggesting no object-based transformation was involved. We also found no significant differences in accuracy or response times between the groups. Nonetheless, SS-E displayed increased response accuracy and consistent response times compared to both groups. On the other hand, GS-E displayed the longest response times, which was consistent with our expectations.

In a second mental rotation task, participants were asked to determine parity (judging whether the figures were the same or different) between a target and a comparison figure using various stimuli such as cubes, poses, letters, and line drawings of hands. The purpose was to evaluate whether this task resulted in an object-based transformation (indicated by a linear trend between response time and angular disparity) and whether the groups exhibited discrepancies based on the stimuli. The significant correlation among response times for cubes, hands, and poses suggests that a parity decision prompted an object-based transformation, specifically for unfamiliar cube figures. However, we cannot detect a significant positive linear trend between response time and angular disparity for letters, indicating that they are not mentally rotated. A group effect is observed in response times and accuracy for cubes, drawings of hands, and soccer-specific poses, with the GS-E group exhibiting advantages. Gymnasts were quicker to perceive all stimuli and had lower response times. Even in soccer-specific poses, gymnasts perceived stimuli faster when presented around various axes. Notably, the results of the experts show that orientation in space is of greater importance than the specific pose. When presented upside down, gymnasts perceive soccer-specific poses faster than soccer players. However, soccer players do not recognize soccer-specific poses faster when presented upside down. Mental rotation of letters is not observed, as no positive linear trend between response time and angular disparity nor any group differences are evident.

Athletes have accumulated significant sensory and motor expertise through years of training and performing diverse activities and skills (O’Regan and Noë, 2001; Blake and Shiffrar, 2007). This expertise leads to neurophysiological and psychological modifications in various body systems, resulting in a sensorimotor system that differs considerably from non-athletes (Tomasino et al., 2013). Thus, mental rotation performance is expected to differ depending on the sensorimotor and psychomotor characteristics of individuals with varying levels of sports-specific expertise. Moreau et al. (2012) instructed their participants to complete a mental rotation test before and after engaging in specific physical training. While mental rotation ability was observed for one activity (wrestling), it was not observed for the other (running). The results indicate that the wrestling group performed better than the running group in the mental rotation test following the physical training. This suggests that adaptations resulting from the training affected mental rotation performance.

Embodied cognition theory asserts that physical movement and motor imagery share a common process, as Wohlschläger and Wohlschläger (1998) explain. Advocates of embodied cognition propose that the organism’s sensory and motor systems are dynamically integrated. This concept is called sensorimotor coupling, which facilitates the efficient use of sensory information during action. Embodied cognitive perspectives have the potential to inform and influence research on motor skills in the domains of sports and sports psychology (Beilock, 2008). Research findings have revealed that the embodiment hypothesis operates in numerous ways in sport-related contexts, including action-specific perception, comprehension, prediction, and decision-making (Calvo-Merino et al., 2005; Casile and Giese, 2006; Moreau et al., 2011, 2012). Action-specific perception, also known as perception-action, is a psychological theory that posits that individuals perceive their environment based on their ability to act (Proffitt, 2006; Witt, 2011). Numerous studies have documented action-specific effects across various contexts (Witt and Proffitt, 2008). The action-specific perception account supports the idea that perception involves processes linking the environment/objects and the perceiver’s capability for action. Similar objects or environments, such as specific soccer poses, including full-span kicks, cross balls, and inside kicks used in passing, appear different depending on the observer’s abilities. As these abilities change over time and with experience, an individual’s perception of comparable objects and environments will similarly change. Soccer players, and especially experts, demonstrate greater accuracy and speed in perceiving single poses, as evidenced by response times that are more consistent and less variable. In contrast, gymnasts exhibit better performance in object-based transformation tasks. These findings corroborate the results of Feng et al. (2017), which showed that sports experts could also make same-different judgements about cubes and hands.

Uttal et al.’s (2013a) taxonomy of spatial abilities is valuable in broadening the perspective that specific sports affect varied visuospatial abilities. The framework emphasizes the intrinsic and extrinsic dimensions of spatial abilities and static and dynamic visuospatial abilities. Extrinsic and dynamic visuospatial abilities are frequently observed in team sports like soccer (Matos and Godinho, 2006). Our exploratory and preliminary study found that soccer players demonstrate improved performance in perceptual tasks due to their better utilization of the visual field in peripheral vision and high binocular visual acuity. In contrast, sports such as gymnastics rely on intrinsic visuospatial abilities that depend on somatosensory information (Pietsch et al., 2019). Thus, this may explain why gymnasts excel in the object-based mental rotation task for all types of stimuli, including soccer-specific poses (see Pietsch, 2018).

### 4.1 Perceptual task with egocentric transformation

We propose that there was no object-based transformation in the initial perceptual task featuring soccer-specific poses. This is because the positive slope criterion of Cohen and Kubovy (1993) was not met. However, numerous studies demonstrate a linear or monotonic increase in reaction time associated with an angular disparity in tasks involving egocentric mental rotation, even though these studies often do not explore the theoretical differences between mental rotation, egocentric transformations, and perspective taking (Kessler and Rutherford, 2010; Jansen and Kaltner, 2014; Kaltner and Jansen, 2014; Voyer et al., 2017; Yu and Zacks, 2017). Based on these findings, it appears that relying solely on a linear correlation between increasing angular disparities and reaction times, as well as considering different instructions or tasks, is insufficient for distinguishing between object-based and egocentric transformations.

Thus, our study’s results do not definitively rule out the possibility of object-based transformation. To successfully complete the mental rotation task in the back view, only a minimal rotation around the body’s longitudinal axis is required to match the orientation of the target pose with one’s own orientation. From the participants’ perspective, mentally moving forward is enough to assume the presented soccer-specific target pose (Steggemann and Weigelt, 2011). While a frontal view of the poses at 80° and 320° angular deviation from the target figure may require an object-based transformation, the back view of the poses at 160° and 240° angular deviation from the target figure does not. The *post hoc* assessment of poses viewed frontally (at 80° and 320° angles) revealed a significantly faster response time (*M* = 1924, SD = 1172) than poses viewed from the back (*M* = 1673, SD = 787.6.4), *t*(55) = 3.61, *p* < 0.01, *d* = 0.251. These findings support Steggemann and Weigelt’s (2011) explanation of an egocentric transformation.

Steggemann et al. (2011) demonstrated that athletes outperformed non-athletes only in mental left-right rotation tasks (egocentric transformations) when considering the effects of expertise and its advantages. Kaltner et al. (2014) demonstrated that sports expertise facilitated performance exclusively for egocentric transformations by eliciting embodied spatial transformations in response to the human body stimulus. However, no evidence of expertise influencing performance in an object-based transformation task with equal-unequal decisions was found. Furthermore, in Feng et al.’s (2017) study on egocentric transformation, participants’ ability to judge body postures was expedited due to their greater familiarity with bodily experiences from a first-person perspective in everyday life. This familiarity potentially enhances performance on egocentric mental rotation tasks. Habacha et al. (2014) discovered a selective effect of motor expertise, as evidenced by the superior performance of sports experts. This was attributed to the increased embodiment of spatial transformations, as indicated by a higher correlation between the mental rotation task and the sports situation. The study affirms a strong association between physical movement and mental execution and identifies the particular aspects of physical activity that affect mental rotation performance. Jola and Mast (2005a,b) conducted tests on both elite dancers and nondancers using a mental body rotation task. They observed no discrepancies between the two groups in their egocentric task involving line drawings of human bodies.

Our exploratory and preliminary study observed No significant group differences in response times. As expected, GS-E and GS-N exhibited the longest response times. Regarding accuracy, a critical factor in decision-making and follow-up actions specific to sports such as soccer, SS-E demonstrated an advantage with an average accuracy rate of 87.7% (compared to GS-E’s 80.8%, SS-E’s 80.8%, and SS-N’s 78.3%). However, the group differences in accuracy, similar to those observed for response times, did not reach significance. This variability in both response time and accuracy is evident in GS-E, GS-N, and SS-N. In contrast, SS-E presents less variance, suggesting a homogeneous group with consistent performance. Gymnasts exhibit lower accuracy rates due to difficulties in identifying soccer-specific poses, resulting in slower decision-making regarding which leg to use for kicking. Egocentric transformations pose a greater challenge for them. Soccer players use the poses to train three skills: full-span kick, cross ball, and inside kick, which are common movements in the game. These skills are observed and practiced during training routines.

### 4.2 Mental rotation task with object-based transformation

The significant associations between the response times of the cubes, hands, and poses indicate that the parity decision induced an object-based transformation. Specifically, we noted a pronounced linear trend in object-based mental rotation for unfamiliar cube figures. These findings align with Kosslyn et al.’s (1998) research, which exhibited a robust object-based transformation for cube figures. The cube figures elicit the longest response times compared to all other stimuli, with the lowest response accuracy. There is a significant difference in response accuracy between gymnasts and soccer players. It is reasonable to assume that sport-specific expertise affects accuracy. Gymnasts possess enhanced spatial ability, which aids in their object-based mental rotation skills, specifically for internally represented objects like cubes, due to the physical requirements of their sport.

Although there seem to be consistent results in mental rotation tasks involving objects such as cubes, findings for human figure rotation are not universally clear-cut and do not exhibit a linear relationship between angular disparity and response time. Parsons observed no linear correlation between response time and angular disparity. The initial research on the mental rotation of figures traces back to Parsons’ 1987 study. He attributed these outcomes to participants’ various techniques to complete the mental rotation tasks. Parsons argued that in contrast to comparing two cubes, mental body rotation necessitates participants to adopt the body position and orientation of the shown stimulus. The study by Zacks et al. (2002) indicates that two types of transformations can be produced depending on the task and instructions. The first type is object-based transformation, which involves mentally rotating objects relative to the reference frame of the environment. The second type is the egocentric, perspective-based transformation, which involves mentally rotating one’s own point of view relative to the object’s reference frame. Due to the linear relationship between angular disparity and response time, we can infer an object-based transformation for the poses analyzed in our research.

Due to the linear relationship between response time and angular disparity for the hand stimuli, it can be inferred that the processing of the task involves object-based mental rotation. Gymnasts outperformed both soccer groups in responding to all hand positions and orientations, which may be attributed to their sport-specific expertise and frequent use of hands. This outcome suggests that motor processes are utilized while performing this task. Response times cannot be assumed to be particularly long when encountering difficulty or inconvenience in positioning their hand to compare the target figure, as the 80° and 320° orientations vary significantly from the 160° orientation but not from the 240° orientation. These findings contrast with the studies conducted by Funk et al. (2005) and Petit and Harris (2005). Both groups of researchers demonstrated that response times were influenced by various factors, including the position of the participant’s own hands, the comfort of the posture depicted, and the difficulty in moving one’s hand into the position of the displayed hand. Additionally, the study revealed that the kinematic constraints of natural motion affect the direction of mental transformation and, therefore, response time trajectories. Response times were affected by both angular disparity and the feasibility of hand movements. This suggests that the body’s motor system plays a role in the mental rotation process when dealing with hand stimuli, distinguishing it from other stimuli types.

In contrast, there is no evidence of mental rotation occurring with letters, as there is no significant positive linear trend between response time and angular disparity. Additionally, the linear increase observed in Cooper and Shepard’s (1978) studies was not as distinct as that observed for cube figures. This finding supports the idea that the mental rotation of objects, like letters, is done using allocentric coordinates, while the mental rotation of body parts, like hands, is done using egocentric coordinates. Another possible explanation for the absence of a linear relationship is that participants may have a higher tolerance for slope when determining the equality of letters (Corballis, 1986). In this regard, the study by Young et al. (1980) demonstrated that neither children nor adults need to mentally rotate letters to identify misaligned letters, as the critical features for recognition are extracted. The visual system detects the objects’ structural properties that remain unchanged despite angular variations. These angle-independent and orientation-independent features help identify the letters’ corresponding components. Once determined, the parity decision can be made without mental rotation.

### 4.3 Limitation, implication, and future direction

It is essential to note the methodological limitations of our research. We could not compare all stimuli directly because cubes and poses are 3D images rotated around three axes, while letters and hands are two-dimensional and rotated on one axis only. As a result, we averaged mental rotation performance across all rotation axes in the respective stimuli instead of showing the function of a single rotation axis. When designing a mental rotation experiment, it is crucial to consider the variety of information processing steps involved and that response time does not reflect the pure “mental rotation time”. Heil (2002) and Jansen-Osmann and Heil (2006) describe analogous sequential processing steps. The mental rotation tasks involve stimulus identification, mental rotation, parity decision, response selection, and motor processes. When discussing response time in mental rotation tasks, it is important to acknowledge that this term represents all processing steps involved. Future studies would benefit from differentiating and analyzing each step to enable detailed statements about the mental rotation process. For a general overview of test design (albeit in relation to psychometric tests and sex differences), see also (Jost and Jansen, 2023). The summarized literature includes comparisons of the stimulus material and the axis of rotation.

The sample size is too small to draw practical and reliable conclusions about the evaluated factors. According to Brysbaert (2019, p. 27), very little research can be adequately conducted with a sample of less than *N* = 100 participants per between-subject group. Brysbaert emphasizes that studies with inadequate sample sizes often fail to detect genuine effects. When effects are observed in such studies, it is usually due to disproportionately large effect sizes within the small samples. Additionally, there is a risk that results that appear to be significant may not actually hold, especially in complex research designs such as the one in the present study. Brysbaert suggests that research with limited participant numbers should focus on topics that can yield reliable results even with a limited amount of data rather than defending studies with too small sample sizes. Specifically, the focus should be on the main effects between two reliably measured within-subject conditions. In this respect, particular caution is required when interpreting the interaction effects within the scope of the study. Additionally, Brysbaert and Stevens (2018) suggest that 216 trials per condition is too low. The authors recommend at least 40 trials per condition to account for the natural variance in reaction times. However, our study only has nine trials per stimulus.

Furthermore, it would have been advantageous to more distinctly separate the groups of soccer-specific experts and novices. Swann et al. (2015) developed a classification system for sports expertise samples, categorizing various types of elite competitive athletes. They propose a method for selecting a valid expert sample. Our exploratory and preliminary study considers variables A, which refers to “highest standard of performance,” and B, which signifies “success at the athlete’s highest level,” in its definition of sports comparison. It distinguishes between soccer players of higher and lower leagues and inquires about their highest sports success. However, the study does not consider variable C, which pertains to “experience at the athlete’s highest level.” Additionally, the variables for comparing sports (D: “Competitiveness of the sport in the athlete’s country” and E: “Global competitiveness of the sport”) were not considered, as all athletes were recruited in Germany.

The chosen soccer-specific poses should have been determined through a consensus of experts. While the current study recognizes a difference in response times between individual poses with egocentric transformation in soccer players versus gymnasts, selecting appropriate poses based on a prior survey would have been beneficial. More accurate results could have been achieved by choosing poses with the highest level of agreement (and recognition).

Specific training can lead to specialized adaptations and improved performance on spatial cognitive tasks. Individuals experiencing difficulties with mental rotation tasks may benefit from this type of training. Concurrently, cognitive training targeting spatial cognition may also improve sport-specific skills. Performance could be further augmented during training breaks or injury interruptions. The precise effects, relationships, and dependencies of these mechanisms are currently unknown, hindering our ability to provide clear recommendations for action.

## 5 Conclusion

In this exploratory and preliminary study, we investigated the mental rotation abilities of gymnasts and soccer players at different levels of expertise. It was found that soccer players, particularly those with high expertise, are faster at recognizing soccer-specific poses when making left-right decisions, indicating a mental rotation process with egocentric transformation. In contrast, our findings show that gymnasts can recognize soccer-specific poses rotated around various axes more quickly and with fewer errors. These results suggest that gymnasts perform faster and more accurately in mental rotation tasks involving object-based transformations, particularly with unknown cube figures, line drawings of hands, and soccer-specific poses. At the same time, no significant differences were observed in the mental rotation of letters. These results suggest that sporting practice and associated motor expertise may enhance the ability for mental rotation, particularly regarding egocentric transformations.

However, it is essential to acknowledge that the preliminary study has methodological constraints due to its small sample size and the limited number of trials per condition. Therefore, the lack of statistical significance in our results does not necessarily indicate the absence of an effect or a relationship between the variables studied. An actual effect may exist but could not be statistically proven due to insufficient sample size and test power. This affects the robustness of the findings and calls for further research with larger sample sizes and increased trials (see Brysbaert, 2019) in mental rotation experiments.

## Data availability statement

The raw data supporting the conclusions of this article will be made available by the authors, without undue reservation.

## Ethics statement

The studies involving humans were approved by the Commission on Responsibility in Research (Ethics Commission) of the University of Stuttgart. The studies were conducted in accordance with the local legislation and institutional requirements. Written informed consent for participation in this study was provided by the participants’ legal guardians/next of kin. Written informed consent was obtained from the individual(s), and minor(s)’ legal guardian/next of kin, for the publication of any potentially identifiable images or data included in this article. All assessments were conducted in accordance with the ethical rules for research in human participants following the Declaration of Helsinki and its later amendments (World Medical and Association, 2015).

## Author contributions

TK: Conceptualization, Data curation, Formal analysis, Investigation, Methodology, Software, Validation, Visualization, Writing – original draft, Writing – review and editing. NS: Conceptualization, Data curation, Formal analysis, Methodology, Validation, Visualization, Writing – original draft, Writing – review and editing.
