# Supplementary material for: Mental rotation abilities of gymnasts and soccer players: a comparison of egocentric and object-based transformations. An exploratory and preliminary study
Source: Front Psychol. 2024 Jun 5;15:1355381. doi: 10.3389/fpsyg.2024.1355381 (PMC11188378; doi:10.3389/fpsyg.2024.1355381)
Supplement: Supplementary file 1 [file Table_1.PDF]

Supplementary material

Soccer-specific poses for the perceptual task with egocentric transformation

**Pose**	**Axis**	**Cross ball**			
	-	80°	160°	240°	320°
Original	Y	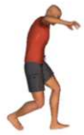	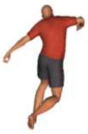	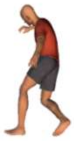	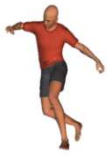
Reflection	Y	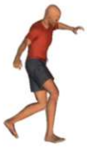	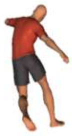	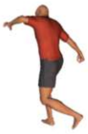	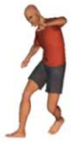
**Pose**	**Axis**	**Inside kick**			
	-	80°	160°	240°	320°
Original	Y	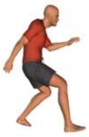	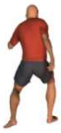	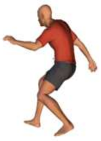	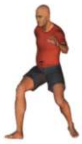
Reflection	Y	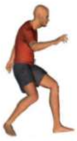	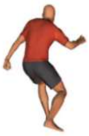	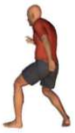	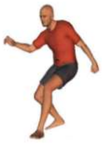
**Pose**	**Axis**	**Full span kick**			
	-	80°	160°	240°	320°
Original	Y	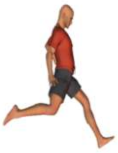	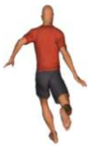	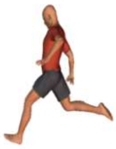	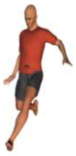
Reflection	Y	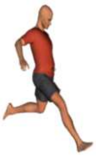	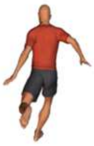	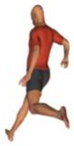	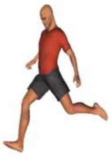

A. Representation of the poses, their reflections, and the 80° - angular orientations around the Y-axis for the perceptual task with egocentric transformation (right-left judgment).

Stimuli for the mental rotation task with object-based transformation

Full span kick	Axis	Target stimuli	Comparison stimuli			
-	-	40°	80°	160°	240°	320°
Original	X	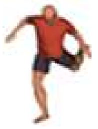	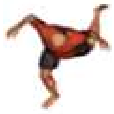	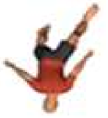	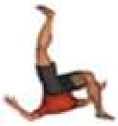	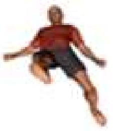
Reflected	X	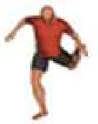	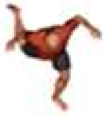	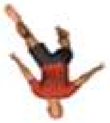	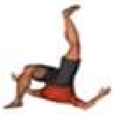	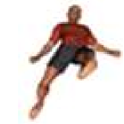

B. Representation of the pose (full span kick), the reflections, and the 80° - angular orientations around the X-axis of the comparison poses. Poses used for the mental rotation task with object-based transformation (same-different judgment). The other two poses and the rotations around the Y and Z axis are not visualized.

Cube 1	Axis	Target stimuli	Comparison stimuli			
-	-	40°	80°	160°	240°	320°
Original	X	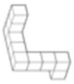	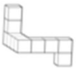	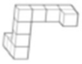	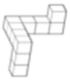	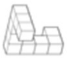
Reflected	X	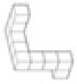	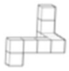	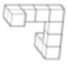	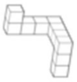	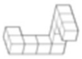

C. Representation of a cube, the reflections, the target cube, and all 80°-angle orientations around the X-axis of the comparison cubes. Cube stimuli are used for the mental rotation task with object-based transformation (same-different judgment). The other two cube figures and the rotations around the Y and Z axis are not visualized.

**Letter G**	**Axis**	**Target stimuli**	**Comparison stimuli**			
-	-	40°	80°	160°	240°	320°
Original	Z	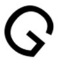	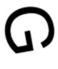	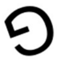	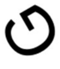	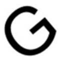
Reflected	Z	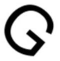	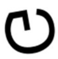	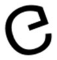	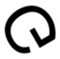	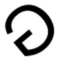

D. Representation of the letter G, the reflections, the target letter, and all 80°-angle orientations around the Z-axis of the comparison letters. Letter stimuli for the mental rotation task with object-based transformation (same-different judgment). The letters J and R are not visualized.

**Hand**	**Axis**	**Target stimuli**	**Comparison stimuli**			
-	-	40°	80°	160°	240°	320°
Original	Z	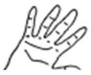	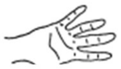	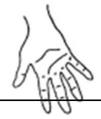	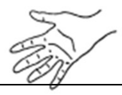	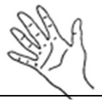
Reflected	Z	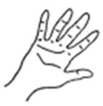	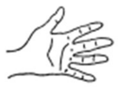	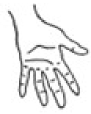	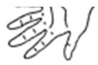	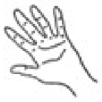

E. Representation of a hand position (palm), the reflections, the target figure, and the 80°-angular orientations around the Z-axis of the comparison hands. Hand stimuli were used for the mental rotation task with object-based transformation (same-different judgment). The other two line drawings of hands are not visualized.

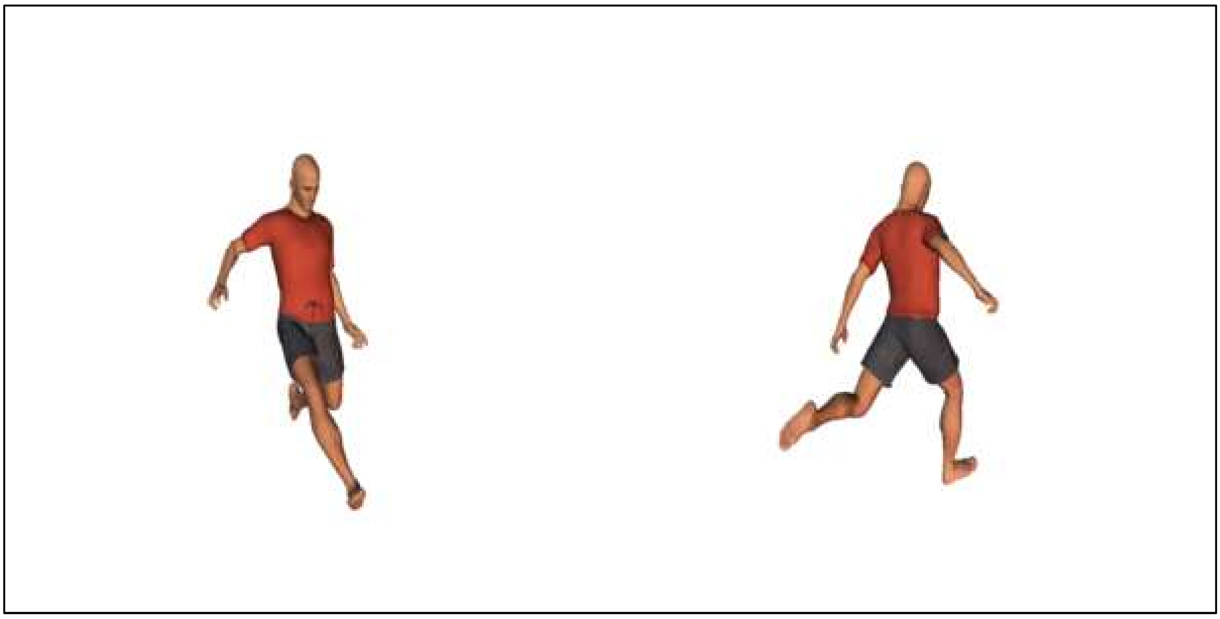

F. Representation of a target pose, and a comparison pose side by side. The task was to decide whether the two poses were identical or not (same-different judgement).
